# Nucleoporin Mediated Nuclear Positioning and Silencing of *HMR*

**DOI:** 10.1371/journal.pone.0021923

**Published:** 2011-07-19

**Authors:** Giulia J. Ruben, Jacob G. Kirkland, Tracy MacDonough, Miao Chen, Rudra N. Dubey, Marc R. Gartenberg, Rohinton T. Kamakaka

**Affiliations:** 1 Department of Molecular Cell Developmental Biology, University of California Santa Cruz, Santa Cruz, California, United States of America; 2 Department of Pharmacology, University of Medicine Dentistry New Jersey - Robert Wood Johnson Medical School, Piscataway, New Jersey, United States of America; 3 Department of Molecular Biology and Biochemistry, Rutgers University, Piscataway, New Jersey, United States of America; Ludwig-Maximilians-Universität München, Germany

## Abstract

The organization of chromatin domains in the nucleus is an important factor in gene regulation. In eukaryotic nuclei, transcriptionally silenced chromatin clusters at the nuclear periphery while transcriptionally poised chromatin resides in the nuclear interior. Recent studies suggest that nuclear pore proteins (NUPs) recruit loci to nuclear pores to aid in insulation of genes from silencing and during gene activation. We investigated the role of NUPs at a native yeast insulator and show that while NUPs localize to the native tDNA insulator adjacent to the silenced *HMR* domain, loss of pore proteins does not compromise insulation. Surprisingly we find that NUPs contribute to silencing at *HMR* and are able to restore silencing to a silencing-defective *HMR* allele when tethered to the locus. We show that the perinuclear positioning of heterochromatin is important for the NUP-mediated silencing effect and find that loss of NUPs result in decreased localization of *HMR* to the nuclear periphery. We also show that loss of telomeric tethering pathways does not eliminate NUP localization to *HMR*, suggesting that NUPs may mediate an independent pathway for *HMR* association with the nuclear periphery. We propose that localization of NUPs to the tDNA insulator at *HMR* helps maintain the intranuclear position of the silent locus, which in turn contributes to the fidelity of silencing at *HMR*.

## Introduction

The regulation of gene expression is governed by the interactions between positive and negative transcription factors and DNA sequence elements. The higher order organization of chromatin domains within the nucleus also contributes to the regulation of gene activity [1]. Transcriptionally active genes adopt a more open chromatin conformation while silenced genes are present within highly inaccessible chromatin domains. These open and closed chromatin domains are not randomly distributed throughout the nucleus but localize to specific regions.

In *Saccharomyces cerevisiae* heterochromatin is established and maintained through the coordinated actions of DNA sequence elements, known as silencers, and repressor proteins [2]. Silencers autonomously initiate silencing by the recruitment of sequence specific factors, which in turn recruit repressor proteins, including the Sir proteins, which deacetylate and bind histones forming a repressed chromatin domain.

Sub-telomeric domains account for the majority of the heterochromatin in yeast. These domains cluster together and are tethered to the nuclear envelope in multiple foci [3], [4], [5] by Ku70/80, Sir4, Esc1 [3], [6], [7] and Mps3 [8]. The clustering of the telomeres at the nuclear periphery thus creates a ‘silent compartment’ in the eukaryotic nucleus [9] located at the nuclear membrane but excluded from the nuclear pores [4], [5], [7] and while nuclear membrane association increases the likelihood of a locus becoming silenced, it is not essential for silencing [10].

The cryptic mating type loci *HMR* and *HML* are also silent in yeast. Repression at these loci is mediated by their cognate silencers, but also depends in part on their proximity to telomeric heterochromatin [11], [12]. *HMR* and *HML* colocalize with telomeric foci at the nuclear periphery [10]. Repression can be restored to a silencer-crippled *HMR* locus by artificially recruiting it to the nuclear membrane, bringing it in spatial proximity to telomeric heterochromatin and the perinuclear-silencing compartment [13].

Numerous reports have recently shown that nuclear pore proteins (NUPs) play a role in regulating gene activity by affecting the intranuclear position of a genomic locus [14]. Numerous NUPs like Nic96, Nup2 and Nup60 physically associate with active genes [15], [16] and it has been shown that some genes become NPC-associated upon activation [15], [17], [18], [19]. The localization of genes at or near nuclear pore complexes (NPCs) has been shown to facilitate efficient transcription [17]. While association with the NPC is not necessary for gene activation, such association maximizes transcriptional activity.

Interestingly, mutations in members of the Nup84 complex have been shown to alter telomere organization [20], [21], [22]. Furthermore, Nup2 is enriched at intergenic regions of sub-telomeric genes and deletion of Nup2, a mobile basket NUP, leads to mild derepression of sub-telomeric loci [23] suggesting a direct or indirect role for NUPs in the regulation of these silenced loci. Taken together, these data provide evidence for a role for NUPs in transcriptional regulation of genes.

Silenced and active chromatin domains reside adjacent to one another and insulator elements are DNA elements that functionally separate them. In yeast barrier insulators restrict the action of silencers, blocking the spread of repression into neighboring active regions. Barriers are often highly active promoters of genes or have promoter characteristics, recruiting various chromatin remodeling and modifying factors to block the spread of silencing [24]. Loss of barrier function, either by mutation of the insulator element or proteins important for insulator activity, results in a spread of silencing into the neighboring region [25], [26], [27], [28]. Barrier activity is commonly monitored by sensitive assays using reporter genes or by fine mapping of Sir proteins across the region in question. Ishii *et al.* showed that ectopic NUP recruitment at a modified silenced *HML* locus could insulate a reporter gene from silencing, and suggested that NUPs functioned as barrier proteins by anchoring the base of chromatin loops to the pore thus separating an active chromatin loop from a silenced chromatin loop [29]. Based on studies with these synthetic constructs it was suggested that recruitment of an insulator element to the nuclear pore complex was a critical step for insulation. Unfortunately the role of NUPs and NPC association at native insulators was not explored in this study.

In this work we investigate the role of NUPs at native insulators in *S. cerevisiae*. We show that NUPs localize to the native tDNA insulator element at *HMR*. Fluorescent microscopy experiments show that loss of Nup60 results in decreased localization of *HMR* at the nuclear periphery, similar to the combined loss of Esc1 and Ku70, while the ectopic tethering of NUPs to a locus recruits that locus to the pore/periphery. However contrary to expectation based on synthetic constructs, NUPs do not function as native barrier proteins in this situation. Our experiments show that NUPs contribute to silencing of sequences adjacent to *HMR*. Furthermore, we find that ectopic recruitment of NUPs to a mutated *HMR* silencer can restore silencing at the locus, likely by the recruitment of this locus to the nuclear periphery. We also find that loss of major heterochromatin tethering pathways does not eliminate Nup60 localization to the tDNA adjacent to *HMR*, suggesting that the NUPs may mediate an independent peripheral positioning pathway for *HMR*. We propose that the contrasting effects of NUPs in both transcriptional activation and repression arise from a context dependent role in the organization and maintenance of chromatin domains at the nuclear periphery.

## Materials and Methods

No human subjects were involved in this research. Only microorganisms were analyzed in this study.

Strain and oligonucleotide details are provided in Table 1 and Supplementary Table S1.

**Table 1:** Primer List.

Probe	Oligo Name	Sequence
		Figure 1
(I) *HMR-I*	Lou168:	5′-GAAGAGACTTATGATCAACATAATTTTGC-3′
	Lou173:	CGCCATATACGAAAATGTTGGTGACATGT-3′
(II) tRNA	Lou201:	5′-CACCAATTCCGCATCTGCAGATTAC-3′
	Lou120:	5′-GGGTGTCACCGAATAACGTGAT-3′
(III) 3′-tRNAa	L108:	5′-TACCGTTATTCGGAGATCTCTTACGG-3′
	L109:	5′-GTGACGCACTGAATGTCATCAAAAG-3′
(IV) 3′-tRNAb	L104:	5′-CATAAGACGAGTTCTTCTATATCCGGT-3′
	L107:	5′-CCTATTTTGCGTATTCCTATGTTG-3′
(V) 5′-*GIT1*	GRO61:	5′-GAGTGTCCGCATGATTAATACTTTTCG-3′
	GRO62:	5′-ATATGAAGATAAATGTGGCACCAAACG-3′
		Supplemental Figure S2
*HMR-I*	Lou168:	5′ GAAGAGACTTATGATCAACATAATTTTGC-3′
	Lou173:	5′-CGCCATATACGAAAATGTTGGTGACATGT-3′
*HMR- tDNA*	Lou201:	5′-CACCAATTCCGCATCTGCAGATTAC-3′
	Lou120:	5′-GGGTGTCACCGAATAACGTGAT-3′
*NL1*	L-143:	5′-CCGGTTTTCTCAAGTTCTGAGCTTCTA-3′
	L-144:	5′-CCATCAGGCATGTTTACCGTAGAATAA-3′
*GR1*	L-139:	5′-AGACAATCCCTTTATGTTTCATGTGCGTA-3′
	L-140:	5′-ATGGATGGCGCGATAATTCTATACC-3′
*KL*	L-187:	5′-AGTATAGCGGAGCCACAAATTTAGCAG-3′
	L-188:	5′-AAATAAAATTTCAAATGCCCTCTGTGG-3′
*ETC9*	L-190:	5′-CAGGAAAATCAAAAGACATGACGCATA-3′
	L-192:	5′-AAAACCGGATAATACCAGGTCAGCTTC-3′
		Supplemental Figure S3
*HMR-E*	L-191:	5′-CCCGTCCAAGTTATGAGCTTAATC-3′
	L-95:	5′-AAAACCAGGAGTACCTGCGCTTATTCT-3′
*HMR-I*	Lou168:	5′-GAAGAGACTTATGATCAACATAATTTTGC-3′
	Lou173:	5′-CGCCATATACGAAAATGTTGGTGACATGT-3′
*HMR*- tDNA	Lou201:	5′-CACCAATTCCGCATCTGCAGATTAC-3′
	Lou120:	5′-GGGTGTCACCGAATAACGTGAT-3′
3′-tRNAa	L108:	5′-TACCGTTATTCGGAGATCTCTTACGG-3′
	L109:	5′-GTGACGCACTGAATGTCATCAAAAG-3′
*TEL7.5*	Roligo 118:	5′-GTGGAAAGTATCGAGTTATGTGTACCT-3′
	Roligo 119:	5′-GTCATTCAAATACAGTGGGAAGTCTAC-3′
*TEL0.5*	Roligo 116:	5′-GACAAATAAAAATTCAGCTTTTTCAAG-3′
	Roligo 117:	5′-GTTCGAATCCTTAAGTAAAACACATTC-3′

Sequence of primers used.

### Plasmids and Transformation of Yeast Strains

GBD fusion plasmids were obtained from the yeast GBD fusion protein collection [30]. In the pGBK-RC-*TRP1* plasmids the *ADH1* promoter drives transcription of the *GAL4* DNA-binding domain (1–147 aa) fused in frame to the N-terminus of different protein. pGBD-*RSC2*, pGBD-*YIP1*, pGBD-*SIR1*, pGBD-*NUP133*, pGBD-*NUP2*, and pGBD-*NUP84* containing plasmids were isolated and transformed into strains JRY4806, TM47 and TM58 as previously described [31].

pJK48 was constructed by cloning the ORF of *NUP133* into a SalI/PstI site of pAT4 resulting in a LexA-Nup133 fusion protein.

### Strain Construction

Strains TM47 and TM58 were derived from the *HMR-E* synthetic silencer strain JRY4806 [32]. The *ΔI* site of JRY4806 was replaced with *URA3* and then *URA3* was replaced by counter-selection with fragments containing the wild-type *HMR-I* silencer generating strain TM58, or a fragment containing a synthetic *HMR-I* silencer containing an Abf1 binding sequence (B) and 5x-Gal binding sequence (5G) to generate TM47. Strains were verified by sequencing.

### Spot Assays

Mating assays were carried out as previously described using tenfold serial dilutions of logarithmically growing cells [31]. When necessary, plasmid selection was maintained throughout the experiment. Cells were grown at 30°C for 2–5 days prior to photography. *URA3* reporter assays were similarly carried out, and spotted onto appropriately supplemented YMD plates to assay for *URA3* expression. Cells were grown at 30°C for 2–5 days prior to photography. In some cases photographs were rearranged to maintain strain order for the purpose of generating clear figures.

### qChIP

Chromatin immunoprecipitation reactions and quantitation were carried out as previously described [27], [33]. Chromatin was sheared by sonication into ∼300-bp fragments and immunoprecipitated using monoclonal anti-c-Myc [9E11] antibody (Abcam, USA). Primer pairs used for quantitative PCR are provided in Table 1.

### Fluorescent Microscopy

Strains GRY 630, 632, 636 and 701 were derived from RDY215 containing a 256x-LacO Array downstream of *HMR* and expressing LacI-GFP for visualization of the locus. Integration of pKW1803 (courtesy of K. Weis, UC Berkeley) introduced *YIPlac*-*dsRED*-*HDEL::NatMX*, a reporter that integrates into the ER lumen (including the space between the inner and outer nuclear envelopes) for visualization of the nuclear membrane. Strains were grown to mid-log phase in Yeast Minimal Media (YMD) plus amino acids W, U, A, L, K, H. Microscopy was carried out on an Olympus iX71 with a DeltaVision stage (Applied Precision) in the GFP and RFP channels at 100X. Images were acquired and analyzed using softWoRx3.7.1 (Applied Precision). Three independent trials were performed for each strain and strains were scored in a blind manner by measuring the distance between the GFP spot (array) and the nuclear membrane (“s2p”) and the diameter of the nucleus (“p2p”) in nanometers. A ratio of (s2p/p2p)*2 was calculated and used for assigning to one of two zones of approximately equal surface area.

#### Localization of *ARS607* with tethered lexA chimeras

Fresh transformants of strain SLJ2594 [8] carrying plasmids expressing LexA (pAT4) or LexA-*NUP133* (pJK48) were grown overnight in synthetic complete media (minus leucine) and sub-cultured to 0.2–0.3 OD_600_ the following morning. Formaldehyde-fixed cells were mounted on slides containing 1.4% agarose plugs. Cells were imaged with a Zeiss Axioplan II fluorescence microscope (100X Plan-Apochromat objective, NA = 1.4) and Axiocam HR camera. Z-stacks were composed of 17 elevations, each separated by 250 nm and an acquisition time of 250 msec. GFP positions were determined according to the method of [3] using the Zeiss Axiovision software package. Measurements were made on three independent trials, as described above, with each cell defined morphologically as G1 or early S. Data for the two different cell stages was combined due to the similarity in values for each.

## Results

### NUPs Localize to a Native Chromatin Boundary in *Saccharomyces cerevisiae*

**Figure 1:**
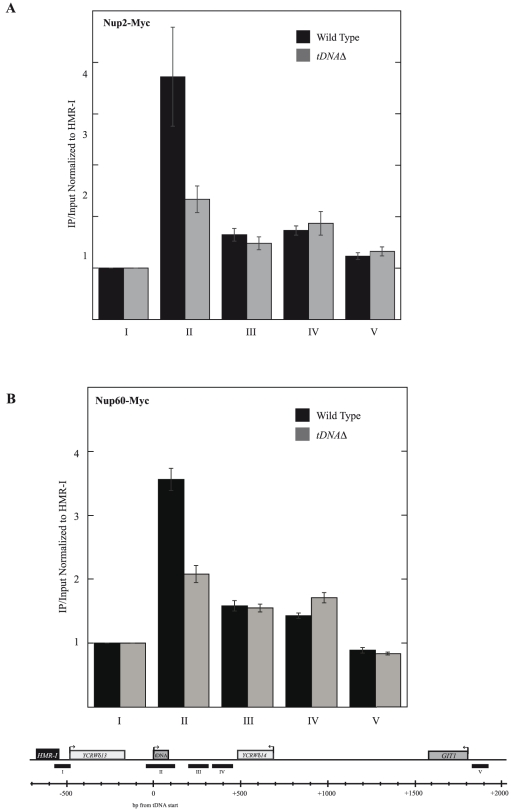
NUPs localize to the *tDNA^Thr^* barrier at *HMR*. Quantitative chromatin immunoprecipitation (qChIP) of Nup2 (A) and Nup60 (B) at *HMR* in wiltd-type and *HMR-tDNA Δ* strains. *Δ*Ct values were derived from the difference in real-time amplification of equal amounts of immunoprecipitated input DNA. Standard error was calculated using data from at least four immunoprecipitation reactions with at least two independently cross-linked chromatin samples. Histogram data was derived by normalizing *Δ*Ct values for the diagramed amplicons against the *Δ*Ct value for the *HMR-I* silencer amplicon (negative control). Both Nup2 and Nup60 show a peak of localization to *tDNA^Thr^* at *HMR* (II), which is greatly reduced in the *HMR-tDNA Δ* strain. (* Represents PCR amplicon V data derived only from two of five independent cross-links for Nup2 qChIP.)

The cryptic mating type locus, *HMR,* is encompassed in a silent chromatin domain and a *tRNA^thr^* gene the barrier insulator that restricts the spread of silencing and insulates the neighboring active genes from repression. In order to determine if the NUPs function as barrier proteins at this native yeast insulator, we initially inquired whether these proteins localize at or near the insulator. Both Nup2 and Nup60 have been implicated as being involved in chromatin domain organization [22], [29]. Nup2 is a mobile nucleoporin, whose localization to the NPC basket is dependent on Nup60 [34]. Quantitative chromatin immunoprecipitation (qChIP) revealed a peak of Nup2 association centered on the tDNA containing probe with a ∼4.5-fold enrichment over the *HMR-I* silencer, which was located ∼600 bp away (Figure 1A). Indeed, Nup2 enrichment was restricted to the tDNA fragment across the entire 2.5 kb region under study. These data indicate that a NUP associates preferentially with the native tDNA barrier. Since Nup2 is a mobile NUP, it was unclear whether the tDNA associates with the nuclear pore or simply with nucleoplasmic Nup2. To address this issue we next performed qChIP for Nup60, a nuclear pore basket NUP. Similar to Nup2, Nup60 also mapped preferentially to the fragment containing the tDNA barrier at *HMR*, ∼3.5-fold over *HMR-I* (Figure 1B).

To determine whether NUP binding was dependent on the tDNA gene or simply coincided with it, the qChIP experiments were repeated in a strain that lacked the internal tDNA promoter. Both Nup2 and Nup60 localization decreased significantly in this strain (Figure 1A and 1B). These data strongly suggest that NUPs are recruited by and localize to the native tDNA barrier at *HMR*. Furthermore, they suggest that sites adjacent to *HMR* associate with NPCs at the nuclear periphery since Nup60, an integral pore NUP found predominantly at the pore, associates with DNA fragment containing the tDNA barrier.

### NUPs Affect the Perinuclear Localization of *HMR*

**Figure 2:**
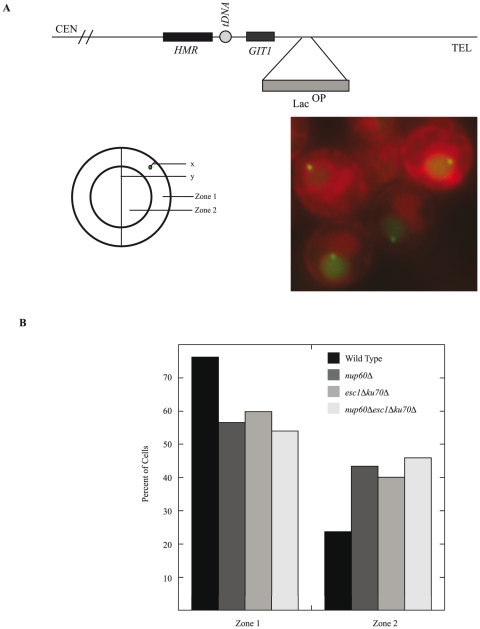
Nup60 affects the perinuclear localization of *HMR*. Fluorescent microscopy was used to visualize the position of a LacO array tagged *HMR* locus (LacI-GFP) with respect to the nuclear periphery (*YIPlac*-*dsRED*-HDEL). The nucleus was divided into two zones of equal surface area (Zone1 = peripheral, Zone 2 = central, see Methods). Percent of cells in zones 1 and 2 were recorded in three separate trials for wild type, *nup60Δ, esc1Δ ku70Δ,* and *esc1Δ ku70Δ nup60Δ* strains (n = 266, 198, 197 and 246 respectively). (A) *HMR* is typically localizes to the nuclear periphery. (B) *HMR* is preferentially localized at the nuclear periphery (Zone 1 = 76.3%). Loss of *NUP60* reduces perinuclear localization equivalent to loss of *ESC1* and *KU70* (Zone 1 = 56.6% and 59.9% respectively, with p values <0.001 by chi square test).

Motivated by the finding that NUPs localize to the tDNA insulator we investigated the relationship between NUPs and native *HMR* localization in the nucleus. We used fluorescent microscopy to test whether NUPs play a role in the peripheral localization of *HMR*. Both *HMR* and *HML* colocalize with telomeric foci at the nuclear periphery [10], [35]. In strains where *HMR* can be excised as an extra-chromosomal ring, it has been shown that loss of *esc1Δ ku70Δ* reduce *HMR's* typical association at the nuclear periphery [10] though the association is not completely abolished. We used a strain with a LacO array placed adjacent to *HMR* in combination with *YIPlac*-*dsRED*-HDEL, a reporter that integrates into the ER lumen including the space between the inner and outer nuclear envelopes, to visualize the nuclear membrane [36], and measure the intranuclear position of *HMR*. The nucleus was divided into two concentric zones of equal surface area and the position of the locus was determined with respect to these zones (Figure 2). The percent of cells in zones 1 and 2 were recorded in three separate trials. We compared wild type, *nup60Δ, esc1Δ ku70Δ,* and *esc1Δ ku70Δ nup60Δ* strains for *HMR's* position with respect to the nuclear periphery. As seen in Figure 2, in wild-type cells *HMR* is localized in zone 1 76.3% of the time while *nup60Δ* reduces *HMR's* position in zone 1 equivalent to *esc1Δ ku70Δ* (56.6% and 59.9%, respectively with p values <0.001 for both). There was a very slight change in the triple mutant (zone 1 = 54.4%) and based on these results we are currently unable to unequivocally state that Nup60 functions in the same or parallel pathway to Esc1 and Ku70. However our results confirm a role for Nup60 in the native localization of *HMR* at the nuclear periphery.

### NUPs do not play a role in native barrier function

**Figure 3:**
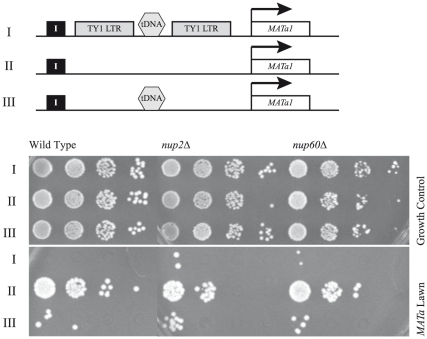
NUPs do not affect boundary function at *HMR*. A phenotypic mating assay for boundary function using a *MATa1* reporter placed downstream of the *HMR-tDNA* barrier. In order from top to bottom: wild-type *tDNA* barrier, complete barrier deletion, and 70-bp *tDNA* barrier with 100-bp flanks. Tenfold serial dilution of overnight cultures with a starting A_600_ of 1.0 was spotted on a fully supplemented minimal medium (growth control) or minimal medium with the mating tester lawn (*MATa* lawn). *MAT* alpha strains expressing the *MATa1* reporter are not able to mate unless boundary function is compromised resulting in repression of *MATa1* (compare first and second rows in wild-type *MATa* lawn panel). *nup2Δ* and *nup60Δ* show no significant differences in boundary function from wild-type.

Because our qChIP experiments revealed that NUPs localize to the tDNA boundary at *HMR,* we wanted to understand whether NUPs function as native barrier proteins. Deletion of the tDNA barrier or proteins that associate with the barrier, such as Rsc, result in an increased spreading of silenced chromatin into the adjacent euchromatic region [25], [26], [27], [37]. If a reporter gene is located in the adjacent euchromatic region, then this reporter becomes transcriptionally silenced. If NUPs function as barrier proteins, we would predict that their absence would result in a loss of barrier function, resulting in the spreading of repression. To test this, we made use of a sensitive phenotypic assay for barrier activity at *HMR*. The native *a1* gene at *HMR* was disabled and an intact copy of the gene was inserted downstream of the native tDNA barrier, as diagrammed in Figure 3. Expression of the gene in a MAT alpha strain creates a pseudo-diploid state that blocks mating and subsequent growth on selective plates. The assay uses three different barrier configurations (Figure 3). In a wild type *MAT* alpha strain the intact barrier blocks the spread of silencing resulting in transcription of *MATa1,* which manifests itself as no growth of the strain on *MATa* containing tester lawns. In the complete barrier-delete strain, barrier activity is lost leading to the spread of silenced chromatin and the subsequent repression of the *MATa1* reporter, which allows the strain to grow on *MATa* tester lawns. Lastly, reinsertion of the 70 bp tDNA barrier, along with 100bp of flanking DNA, is sufficient to rescue barrier function. Analyses of strains with these barrier constructs but lacking either Nup2 or Nup60 were performed, and we did not observe any significant difference in barrier activity between wild type and mutant strains. These results demonstrate that while both Nup2 and Nup60 are present at the *HMR* boundary, and help recruit the locus to the periphery, they do not play a significant role in native barrier function. In addition, no change in the distribution of silencing protein, Sir3, was seen by qChIP past the tDNA barrier region in a *nup2Δ* strain (Supplementary Figure S1).

### NUPs affect silencing

**Figure 4:**
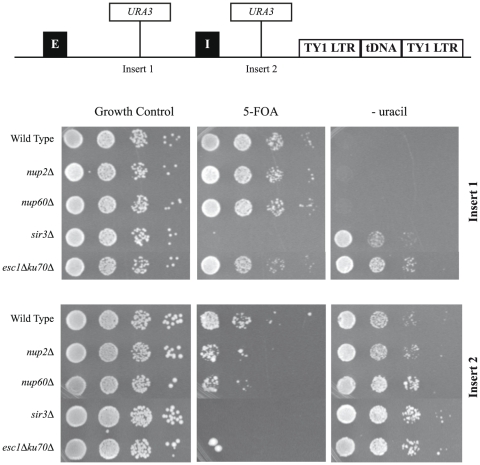
NUPs contribute to the maintenance of silencing near the boundary at *HMR*. A phenotypic assay for analyzing the silent state at *HMR* using a *URA3* reporter gene located either between the *HMR-E* and *HMR-I* silencer (Insert 1) or between the *HMR-I* silencer and the *tDNA^Thr^* barrier (Insert 2). Combined with *nup2Δ*, *nup60Δ, sir3Δ* or *esc1Δ ku70Δ,* spot dilutions were performed as described in Figure 2. Strains were tested on counter-selection media containing 5-Fluoroorotic acid (5′-FOA) or media lacking uracil. Strains are unable to grow on 5′-FOA unless the *URA3* reporter is stably repressed over multiple generations. Insert 1 combined with *nup2Δ* or *nup60Δ* behaves just as wild type, confirming stable repression of *HMR*. *sir3Δ* expectedly exhibits complete loss of silencing at *HMR*, unable to grow 5′-FOA. *esc1Δ ku70Δ* reveals metastable silencing, growing both on 5′-FOA and on media lacking uracil. Insert 2 shows a moderate loss of growth on 5′-FOA in *nup2Δ* and *nup60Δ* strains, suggesting that both *nup2Δ* and *nup60Δ* disturb the maintenance of silencing at *HMR*. *esc1Δ ku70Δ* shows robust loss of silencing, switching the metastable silent state in wild type strains to completely de-repressed, as seen by no significant growth on 5′-FOA.

Localization of genes to the nuclear membrane often leads to their repression [13]. Since we find NUPs adjacent to the silenced *HMR* domain, and since NUPs affect the peripheral localization of *HMR*, we asked if loss of NUPs affected silencing of the *HMR* locus. We performed a sensitive assay for loss of silencing, which monitors expression of a *URA3* reporter gene by growth on media containing 5-Fluoroorotic acid (5′-FOA), which requires the stable silencing of *URA3* over many generations to grow [31], [38]. A *URA3* reporter gene under it's own promoter was placed either between the *HMR-E* and *HMR-I* silencer or between the *HMR-I* silencer and the *tDNA^Thr^* barrier as diagrammed in Figure 4. In the first construct (Insert 1) the wild type strain grows robustly on 5′-FOA and exhibits no growth on media lacking uracil, as expected for complete repression of *URA3* within the silent domain at *HMR*. Both *nup2Δ* and *nup60Δ* do not affect maintenance of the silent state at *HMR* in this construct. Strains lacking Sir3p (*sir3Δ)* exhibit complete loss of silencing, with no growth on 5′-FOA but growth on media lacking uracil, as expected. In the second construct (Insert 2) *URA3* was inserted between the *HMR-I* silencer and the tDNA barrier. These strains exhibit growth on both 5′-FOA containing media as well as media lacking uracil. This result suggests that the border of the silent domain at *HMR* exists in a metastable silencing state. In both the *nup2Δ* and *nup60Δ* strains, growth is slightly reduced on 5′-FOA containing plates. These results suggest that silenced chromatin is slightly perturbed upon deletion of *NUP2* or *NUP60*. The slight effect is consistent with RT-PCR measurements where we were unable to detect any significant difference between wild-type and mutant strains (data not shown). Given the magnitude of this effect, it could be due to direct or indirect effects such as redistribution of Sir proteins or alterations in nuclear architecture or other mechanisms.

### Recruitment of NUPs to *HMR* Establishes Silencing

Given the published data demonstrating that NUP mediated tethering of genes to the nuclear pore aids in gene activation and given the fact that loss of NUPs only weakly affects silencing it was not clear if the observed effect on silencing was direct or indirect. However our data clearly show that NUPs bind the tDNA and aid in recruitment of the *HMR* domain to the nuclear periphery/pore. We therefore asked what is the consequence of artificially recruiting pore proteins to the *HMR* locus specifically. We asked whether the recruitment of specific NUPs to a mutant silencer would overcome the silencing defect associated with the mutant silencer and restore silencing at the compromised locus.

**Figure 5:**
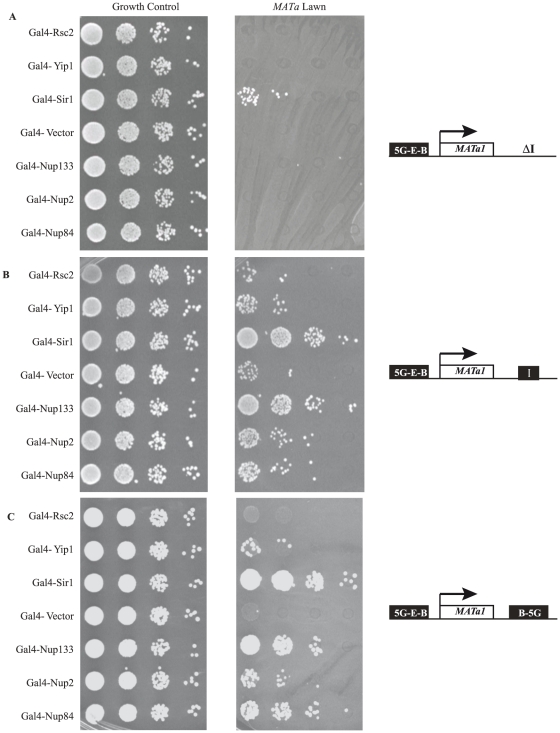
NUP-tethering of *HMR* to the nuclear periphery can establish silencing. A phenotypic assay for the establishment of silencing at *HMR* using three different synthetic silencer constructs for targeted recruitment of Gal4-fusion proteins: (A) *HMR-E*::5G-E-B (G-Gal binding sequence, E-Rap1 binding sequence, B-Abf1 binding sequence) lacking *HMR-I,* (B) *HMR-E*::5G-E-B with *HMR-I,* and (C) *HMR-E*::5G-E-B *HMR-I*::B-5G. The endogenous *MATa1* gene serves as the reporter. All three constructs alone result in de-repression at *HMR*, and an inability to mate on *MATa* lawn (row 4, all panels). Strains were transformed with Gal4-fusion proteins: Gal4-Rsc*2*, Gal4-Yip1, Gal4-Sir1 (positive control), Gal4-Nup133, Gal4-Nup2, Gal4-Nup84 and Gal4-Vector control. Transformed strains were grown under selection to maintain plasmid (media lacking tryptophan) and spot dilutions were performed as described in Figure 2. Re-establishment of repression at *HMR* results in growth on *MATa* lawn, as evidenced by Gal4-Sir1 rescue (row 3, all panels).

There are two silencers flanking the native *HMR* locus called *HMR-E* and *HMR-I*. *HMR-E* is essential for silencing while *HMR-I* is important for silencing but significant levels of silencing are observed in the absence of *HMR-I*. The *HMR-E* silencer contains binding sites for ORC, Rap1 and Abf1 while the *HMR-I* silencer contains binding sites for ORC and Abf1 [2]. A strain lacking *HMR-I* and containing a mutation in one of the sites in *HMR-E* is unable to silence reporter genes. Similarly, an *HMRΔI* strain where Gal4 binding sites replace the ORC sites at *HMR-E* is unable to silence a reporter gene since this silencer is also unable to bind ORC and so is unable to recruit Sir1. However expression of Gal4-Sir1 in this strain restores silencing to some extent since this fusion protein can bind the Gal4 sites in the synthetic *HMR-E* silencer [32]. Using this strain we asked if tethering other proteins could also restore silencing. We tested various proteins; Gal4-Rsc2, a chromatin remodeler that is recruited to tDNAs including the *HMR* barrier tDNA [27], Gal4-Yip1, an integral membrane protein, which has previously been shown to silence a derepressed allele of *HMR* by recruitment to the nuclear periphery [13], and several NUPs fused to Gal4. While Gal4-Sir1 restores silencing in the *HMRΔI* background containing the synthetic *HMR-E* (GEB) silencer [32], none of the other proteins tested when recruited to this synthetic *HMR-E* silencer were able to silence the reporter gene (Figure 5A).

The *HMR-I* silencer communicates with *HMR-E* and acts as a proto-silencer and aids in the stable repression of reporter genes [39]. We next asked if artificial tethering of fusion proteins – Gal4-Rsc2, Gal4-Yip1, and the Gal4-NUPs could aid in silencing in the presence of wild type *HMR-I* (figure 5B). The presence of *HMR-I* restores some silencing even in the absence of Sir1 (see vector alone) confirming its proto-silencer activity. In combination with *HMR-I,* very robust rescue of silencing is observed for Gal4-Sir1, and significant silencing is observed in the presence of Gal4-Yip1 and the different Gal4-NUPs tested. Gal4-Nup133 was most robust in silencing while Gal4-Nup84 and Gal4-Nup2 were not as robust in silencing though they were equivalent to Gal4-Yip1 in their ability to silence (Figure 5B) while Gal4-Rsc2 was not able to increase silencing at this locus. Thus the presence of *HMR-I* enables specific fusion proteins to silence a reporter gene when ectopically recruited to the synthetic *HMR-E* (GEB) silencer. These data indicate that the NUPs have the ability to increase silencing when recruited to a sensitized silenced domain.

We next tested a synthetic silencer construct where Gal4 binding sites replaced all of the ORC sites at both the *HMR-E* (GEB) and *HMR-I* (GB) silencers. Similar to the previous results, Gal4-Nup133 was most robust in silencing while Gal4-Nup84 and Gal4-Nup2 were able to silence as well if not better than Gal4-Yip1 (Figure 5C). These data indicate that NUP-mediated silencing is dependent on the presence of either native *HMR-I* or flanking the reporter by NUPs.

**Figure 6:**
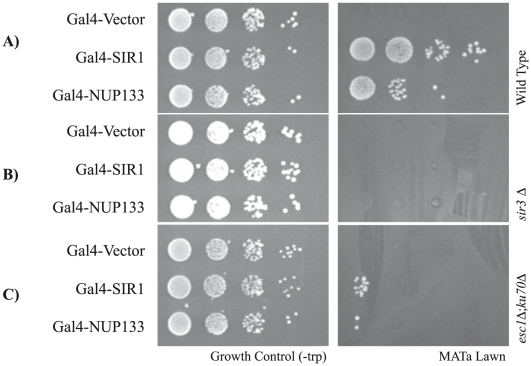
NUP-tethered silencing at *HMR* requires Sir3 and peripheral membrane proteins, Esc1 and Ku70. Testing of NUP-tethered silencing phenotype in *sir3Δ* and *esc1Δ ku70Δ* backgrounds. Synthetic silencer strain *HMR-E*::5G-E-B *HMR-I*::B-5G (Figure 5C) was transformed with Gal4-Sir1, Gal4-Nup133 or Gal4-Vector and tested as described in Figure 5. A) Strains carrying Gal4-Sir1 and Gal4-Nup133 establish silencing. (B) Sir1- and Nup133-mediated silencing is abolished in silencing defective *sir3Δ* background. (C) Nup133-mediated silencing is dependent on Esc1 and Ku70.

We tested whether NUP-tethered silencing was dependent on the ability of a cell to establish heterochromatic domains, as it might be possible that NUP-tethering affected reporter activity via another regulatory pathway. To this end we repeated our assay in the synthetic silencer construct where Gal4 binding sites replaced ORC sites at both the *HMR-E* and *HMR-I* silencers, and transformed the strains with Gal4-Sir1 and Gal4-Nup133 (Figure 6A). As expected, in a silencing deficient *sir3Δ* background, neither Gal4-Sir1 nor Gal4-Nup133 was able to restore silencing (Figure 6B). Similar results were obtained in a *sir2Δ* strain (data not shown) confirming that the NUP-mediated silencing effect was in fact operating through the silencing pathway.

### NUPs position a chromosomal locus to the nuclear periphery

**Figure 7:**
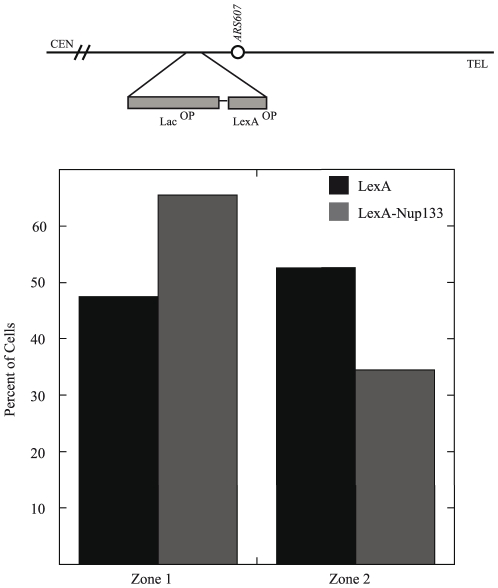
NUPs redirect an internal locus to the nuclear periphery. Fluorescent microscopy was used to visualize an internal LacO array located at *ARS607* on Chr. VI [3]. LexA binding sites next to the LacO array allow recruitment of LexA (pAT4) or LexA-Nup133 (pJK48) to the locus. Position of the array was visualized by LacI-GFP and measured with respect to the periphery using *NUP49*-GFP to mark the nuclear membrane. The nucleus was divided into two zones of equal surface area (Zone1 = peripheral, Zone 2 = central, see Methods). Percent of cells in zones 1 and 2 were recorded in three separate trials (n = 228 for pAT4, n = 226 for pJK48). LexA alone shows random distribution in the nucleus (Zone 1 = 47.4%, Zone 2 = 52.6%). LexA-Nup133 recruitment increases percentage of cells with *ARS607* in Zone 1 to 65.5% showing Nup133 is able to redirect this locus to the nuclear periphery (p<0.001 by chi square test).

Since we observed that NUPs affected native silencing and positioning of *HMR,* in addition to restoring silencing to a silencer-crippled *HMR*, we hypothesized that NUPs contribute to silencing at *HMR* by contributing to the position of the *HMR* domain at the nuclear periphery. One prediction of this model would be that recruiting NUPs to a locus should lead to the repositioning of that locus to the nuclear envelope/pore. Given our NUP-mediated silencing result with Gal4-Nup133, we wanted to verify that NUP recruitment to a genomic locus could redirect its position toward the nuclear periphery. To this end, we used fluorescent microscopy to visualize a LacO array located at *ARS607* on Chr. VI. This locus is randomly positioned in the nucleus [3]. LexA binding sites inserted next to the LacO array allow recruitment of a LexA-fused protein to the locus, similar to our Gal4-NUP-mediated silencing assay. These strains were transformed with a LexA vector (pAT4) or LexA-*NUP133* (pJK48) expressing plasmid. The position of the array was measured with respect to the periphery using *NUP49*-GFP to mark the nuclear membrane. The nucleus was divided into two concentric zones of equal surface area and the position of the locus was determined with respect to these zones. The percent of cells in zones 1 and 2 were recorded in three separate trials (Figure 7). As shown previously [7], the LexA plasmid alone showed a fairly random positioning of *ARS607* in the zones (Zone 1 = 47.4%, Zone 2 = 52.6%). Recruiting LexA-Nup133 to the locus increased the localization of *ARS607* to Zone 1 to 65.5% (p value <0.001). Similarly, it has been shown [7] that Gal4-Yif1 (a nuclear membrane protein) increases the localization of *ARS607* from the interior to the periphery in line with our Gal4-Nup133 results. This verifies that NUPs can bias a locus to the nuclear periphery, as has been previously suggested [29].

### Perinuclear Architecture of Silent Heterochromatin is Important for NUP-Mediated Silencing

Esc1 and Ku70 are membrane-associated proteins that interact with Sir4 and are important for the anchoring of telomeres and *HMR* to the nuclear periphery. Loss of Esc1 and Ku70 results in both the release of telomeres from the periphery as well as the dispersion of Sir proteins from perinuclear clusters [3], [7], [40], [41]. We repeated our NUP-targeting assay for silencing using Gal4-Nup133 in an *esc1Δ ku70Δ* background. As seen in Figure 6C, the rescue of silencing at *HMR* by Gal4-Sir1 and Gal4-Nup133 is dependent on the presence of Esc1 and Ku70 in the cell. This result supports the model that the heterochromatic anchors Esc1 and Ku70 are important for the NUP-targeting silencing effect. In addition, silencing mediated by ectopic positioning of *HMR* to a nuclear compartment enriched for Sir proteins is the most likely molecular mechanism underlying NUP-targeted silencing. To extend this finding to the native *HMR*, we repeated our *URA3* silencing assay in an *esc1Δ ku70Δ* background. As seen in Figure 4, loss of Esc1 and Ku70 results in a change to a metastable silent state when *URA3* is placed between the *HMR* silencers (Insert 1) and complete de-repression when *URA3* is located between the *HMR*-*I* silencer and the tDNA barrier (Insert 2). This result suggests that the heterochromatin environment mediated by Esc1 and Ku70 at the nuclear periphery is important in the native maintenance of silencing at *HMR*.

### NUPs may mediate an independent pathway for *HMR* positioning at the nuclear periphery

**Figure 8:**
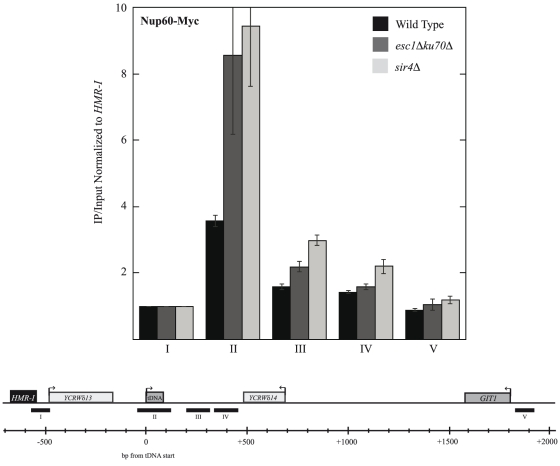
NUP localization to the tDNA increases in the absence of telomeric tethering pathways. qChIP for Nup60 was performed in *esc1Δku70Δ* and *sir4Δ* backgrounds where heterochromatin tethering pathways are disrupted. Amplicons used were identical to those in Figure 1 (see schematic in Figure 1). Loss of *ESC1* and *KU70*, or *SIR4*, appears to increase Nup60 localization to the tDNA barrier (p = 0.08 and p = 0.05, respectively).

Our results so far led us to hypothesize that NUPs may mediate an independent, pathway for tethering to the nuclear periphery. In order to better understand how perinuclear architecture of heterochromatin affected NUP localization near *HMR*, we repeated our qChIP experiments in *esc1Δ ku70Δ* and *sir4Δ* backgrounds. Both Sir4 and Esc1/Ku have been previously shown to aid in the clustering and tethering of heterochromatin to the nuclear periphery [10], [41], [42]. Strikingly, loss of *SIR4* or *ESC1* and *KU70* appears to increase the localization of Nup60 to the tDNA barrier (Figure 8). qChIP for Nup60 in these strain backgrounds is variable, likely due to the disruption in perinuclear architecture in these mutants [43]. Together with our results showing that Nup60 remains localized at the *HMR* tDNA in the absence of silencing (Supplementary Figure S3), our data suggest that sequences adjacent to *HMR* can associate with NPCs independently of canonical heterochromatin tethering pathways, and that in their absence, *HMR* is biased toward the NPC-mediated pathway due to the association of NUPs with the tDNA resulting in an apparent increase in Nup60 localization near *HMR*.

## Discussion

Unlike recent reports in yeast, *Drosophila,* and humans where NUPs have been found to play a role in transcriptional activation, our study aims to elucidate the role of NUPs in insulation and silencing. We show that nuclear pore proteins localize to a native tDNA barrier at *HMR,* and weakly contribute to the maintenance of silencing at *HMR*. In addition, we find a rescue of silencing upon recruitment of NUPs to a derepressed *HMR* locus. Microscopy data confirms that NUPs are able to redirect a genomic locus to the nuclear periphery, and that Nup60 is involved in maintaining the native perinuclear position of *HMR*. In addition, we find that NUP localization at the tDNA adjacent to *HMR* is independent of silencing, and the major heterochromatin tethering pathways mediated via Sir4, Esc1 and Ku, suggesting that NUPs may mediate an independent pathway for the peripheral localization of *HMR*.

### Are NUPs genuine barrier proteins?

Active and silenced domains are often juxtaposed, and mechanisms have evolved to stably separate these two antagonistic chromatin states. Insulators are DNA elements that functionally separate active from silenced chromatin domains. In yeast most insulators are promoters of specific genes that also function as insulators via the recruitment of chromatin modifiers and remodelers, while in metazoans insulators are autonomous elements, but also utilize chromatin modifiers for insulation [24], [44]. In addition, insulators often associate with the nuclear periphery or cluster at specific sites in the nucleus and it has been proposed that this localization might play a crucial role in insulation [45].

Not much is known about the nuclear localization of native *S. cerevisiae* insulators. In order to identify proteins involved in yeast insulation, a synthetic screen for insulator proteins was performed using a dual reporter cassette at a modified *HML* locus [29]. In this screen nuclear transport factors and pore proteins, like Nup2, were identified as “genuine barrier” proteins because they specifically insulated the *ADE2* reporter from repression while maintaining the neighboring *URA3* gene in a silent state. In these experiments, factors with barrier activity (BA) were distinguished from transcriptional activators and desilencing activities, thus termed “genuine barrier” proteins. Based on these results it was proposed that tethering of insulators to the pore resulted in the formation of topologically distinct chromatin loops, separating active gene loops from silenced loci, and that tethering of a locus to the nuclear pore was the key mechanism of insulation. However, the study did not determine whether NUPs localized to native insulators or whether they had any barrier function at native insulators. In this study, we have shown that NUPs do in fact localize to the native tDNA barrier element at the *HMR* boundary, but found no evidence to support their function as native barrier proteins. On the contrary we found that loss of Nup2 or Nup60 caused decreased silencing in the region between the *HMR-I* silencer and the tDNA barrier, as opposed to the spread of silencing which would be predicted if NUPs were native barrier proteins. Our current results suggest that the phenotypic readout in the boundary trap assay used by *Ishii et al*. was probably due to the ability of Nup2 to function as a proto-silencer leading to increased silencing of basal *URA3* expression (as monitored by growth on 5-FOA) [46] while not affecting Bas1 and Pho2 mediated activation of *ADE2* (as monitored by growth on media lacking adenine) [47]. This possibility is further supported by our subsequent findings that NUPs are able to increase silencing in a Sir dependent manner when recruited to synthetic silencers at *HMR*.

### Contrasting roles for NUPs in gene regulation

In budding yeast, most active genes reside in the interior of the nucleus while inducible genes localize to the nuclear pores upon induction [14]. The factors required to recruit genes to the pores are being elucidated and only specific activators and coactivators are recruited to the pore. For example, the SAGA complex involving Ada2, Sus1 and the THO-TREX complex, along with Mex67, aid in recruiting genes to the pore via interactions with Nup1 [48], [49], [50], [51]. An alternative pathway utilizes the histone variant H2A.Z and Nup2 for recruitment. While tethering of genes to the pore is not necessary for gene activation, tethering allows rapid reactivation of the gene thus acting as a form of epigenetic memory for the gene state [17], [52].

In contrast to gene activation, silenced loci are usually found localized at the nuclear membrane [53]. The thirty-two telomeres are found in less than ten foci in the nucleus and multiple telomeres cluster together along the nuclear periphery [5]. While nuclear membrane tethering is not necessary for silencing [10], tethering increases the stability of the silent state by recruiting the silenced loci to a nuclear compartment rich in silencing proteins [13]. The tethering of telomeres to the membrane involves multiple redundant pathways. Esc1 is a membrane protein that anchors telomeres to the membrane via interactions with Sir4 [7]. An alternative pathway involves Ku that anchor chromosomes to the membrane independently of the Sir proteins via interactions with telomerase subunits and the SUN domain protein Mps3 [54]. A third pathway involves the nuclear pore proteins- specifically the Nup84 complex (Nup84, Nup120, Nup133 and Nup60) [55]. Immunofluorescence analyses demonstrate that telomere tethering to the nuclear periphery is altered in Nup84 nuclear pore complex mutants [20] and this functions through Slx5, Slx8 and Ulp1[56].

These apparently contradictory observations, that silent heterochromatin resides at the nuclear membrane and is excluded from nuclear pores [3], [4], [5], [6], [7], [8] while active genes reside at the pores [17], [57], can be reconciled based on our results. We show that the tRNA gene adjacent to the silenced *HMR* locus is bound by NUPs, and that NUP association plays a role in the tethering of the *HMR* locus to the nuclear periphery. Comparably, genome wide mapping data shows a large number of active genes that associate with NUPs are in fact sub-telomeric genes [15], [23]. It is imaginable that interactions of active sub-telomeric genes with the NPC could aid in their activation and simultaneously play a role in telomere tethering to the nuclear periphery, and thus loss of tethering to the pore could affect telomere clustering at the periphery. At this point it is not clear whether transcription of the tDNA and sub-telomeric genes is necessary for peripheral tethering. On that note, the tDNA insulator at *HMR* is transcriptionally active [25], and a transcriptionally inactive tDNA pseudogene, *ETC9*- that functions as a weak insulator [33], does not bind Nup2 (Supplementary Figure S2). The association of NUPs with the tDNA barrier at *HMR* is consistent with immunoelectron microscopy that shows the tRNA transcription factor TFIIIC localizes to the nuclear pore and co-immunoprecipitates with nuclear transport proteins [58].

Our demonstration that loss of Nup2 or Nup60 reduces silencing (albeit weakly) at *HMR* is also consistent with the fact that while localization at the periphery is not absolutely essential for silencing it aids in the stabilization of the silent state. Our data suggest that, paradoxically, this is achieved in part by the tethering of active genes to the nuclear pore, the consequence of which is to position adjacent sequences near the silencing compartments rich in Sir proteins, thereby increasing silencing at these loci. We show that if a reporter gene is near a canonical silencer and weakly repressed, placing a secondary element recruiting the locus to the nuclear periphery, be it the nuclear pore or the nuclear membrane, increases silencing of the reporter gene. Conversely, if a reporter gene is transcriptionally active and recruited to a nuclear environment rich in gene activators, like the NPC, then this relocalization aids in gene activation [17]. It is known that peripheral localization is not required for either silencing or activation, but does affect the efficiency and heritability of the process. While these effects may be subtle on a lab time scale, they may affect organism fitness and have significant consequences in evolutionary time scales.

Insulators block the action of long-range regulatory elements such as enhancers and silencers. It is believed that these elements are neutral elements in that they do not directly affect the activity of enhancers or silencers. Our data suggest that while native insulators do not directly affect silencers, they can affect the chromatin environment-both positively and negatively. The DNA sequences immediately adjacent to the tDNA insulator are nucleosome free and adopt an open chromatin state due to the recruitment of chromatin remodelers and modifiers [26], [27], [59]. However, at the same time, tethering of the tDNA insulator to the NPC results in the recruitment of chromatin to the nuclear periphery rich in Sir proteins. Consequently, silencing of the chromatin between the silencer and the insulator is strengthened. In the future, insulator activity measurements based on the expression of reporter genes should be carefully evaluated with particular regards to flanking regulatory elements, be they enhancers or silencers. This may also help explain the complexity of effects of mutations in insulators, enhancers and PRE elements at the *Drosophila* bithorax locus.

### NUPs contribute to chromatin domain organization at the nuclear periphery

In yeast both the telomeres and *HM* loci localize to the nuclear periphery, creating a perinuclear environment enriched for silent heterochromatin [35]. Localization of heterochromatin is dependent on two membrane-associated proteins Esc1 and Ku70. Esc1 interacts with Sir4 while Ku interacts with telomerase and the *HMR* and *HML* silencers [7], [60], [61], [62]. Mutations in these two proteins cause loss of perinuclear positioning of silenced loci as well as a dispersion of Sir proteins from the nuclear periphery [41]. Indeed we confirm that loss of Esc1 and Ku70 reduce the peripheral association of *HMR*. We show here that NUPs contribute to the intranuclear position of *HMR*, likely via the tDNA barrier element. We cannot conclude that NUPs independently position *HMR* at the periphery based simply on our microscopy results, but our ChIP results suggest this may be possible. Interestingly, the binding of Nup60 at the tDNA adjacent to *HMR* is independent of silencing, as it's binding profile is unchanged in a *sir3Δ* background (Supplementary Figure S3) suggesting that *HMR* associates with the NPC independently of silencing. Furthermore, despite the fact that *sir4Δ* and *esc1Δ ku70Δ* result in a loss of perinuclear positioning of heterochromatin as well as a dispersion of Sir proteins from the nuclear periphery, *HMR*'s interaction with NPCs seems to stand out, as evidenced by increased localization of Nup60 to the tDNA at *HMR*. Thus, it is likely that multiple independent pathways play a supportive role in *HMR* positioning, including NUP association with the tDNA, and thus contribute to the overall regulation of chromatin at *HMR*. We hypothesize that in the absence of canonical heterochromatin-anchoring pathways, *HMR* is biased toward an NPC-mediated anchoring pathway to remain at the periphery, and possibly maintain its epigenetic state. In effect, the role of each independent pathway may not be critical, but taken together help elucidate the evolution of intricate pathways that have converged to maintain strict control over gene regulation.

Clearly, silent and active chromatin domains coexist at the nuclear membrane, and numerous pathways come together contributing to this organization of chromatin. The regulation of distinct functional domains at the nuclear periphery is an intriguing topic as it posits that intranuclear positioning plays an important role in the regulation of chromatin domains. Our study, along with others, suggests that the equilibrium between functional chromatin domains at the nuclear periphery is mediated, in part, by NUPs. Furthermore, our findings reconcile conflicting evidence for a role for NUPs in both activation and repression by putting forward the model that NUPs influence the regulation of gene expression depending on genomic context. In the case of *HMR*, it happens that NUP association with the active tDNA insulator element also serves to maintain the intranuclear position of *HMR,* in turn strengthening silencing.

## Supporting Information

Figure S1 **Loss of** ***NUP2*** **does not affect the distribution of Sir3 at** ***HMR.*** qChIP for Sir3 at *HMR* in wildtype and *nup2Δ* strains. Histogram data was derived by normalizing ΔCt values for the diagramed amplicons against the ΔCt value for the *TEL7.5* amplicon, a negative control located 7.5 kb from *TEL6R* where Sir3 is depleted. *TEL0.5* amplicon is located 0.5 kb from *TEL6R*, and serves as a positive control. *nup2Δ* does not affect the distribution of Sir3 at the *HMR* boundary.
(EPS)

Figure S2 **NUPs localize to tDNAs irrespective of barrier activity.** qChIP of Nup2 was performed as described in Figure 1 for other yeast tDNA genes previously tested for barrier activity: *tRNA^Thr^ NL1* (Chr XIV), *tRNA^Thr^ KL* (Chr XI), *tRNA^Thr^ GR1* (Chr VII) and pseudo *tRNA^Arg^ ETC9* (Chr VII) {Valenzuela, 2008 #4375}. Nup2 shows enrichment at the other tDNAs, with and without barrier activity, except at *ETC9* which does have barrier activity.
(EPS)

Figure S3 **NUPs localize to the tDNA barrier at** ***HMR*** **in the absence of silencing.** qChIP for Nup60 was performed in a *sir3Δ* background where heterochromatin cannot be established. Amplicons used were identical to those in Figure 1 (see schematic in Figure 1). Nup60 localization in not significantly altered in the absence of silencing (p = 0.13).
(EPS)

Table S1 List of strains used and their associated genotypes.
(DOCX)
