# Supplementary material for: Nucleoporin Mediated Nuclear Positioning and Silencing of HMR
Source: PLoS One. 2011 Jul 19;6(7):e21923. doi: 10.1371/journal.pone.0021923 (PMC3139579; doi:10.1371/journal.pone.0021923)
Supplement: Table S1 — List of strains used and their associated genotypes. (DOCX) [file pone.0021923.s004.docx]

**Supplementary Table S1:**

Strain Table. All strains are isogenic to W303.

| **Strain No** | **Genotype** |
| --- | --- |
| GRY104 | *MATa ade2-1 LYS2 HMRaΔp- (TY1-tRNA-TY1) - a1* |
| GRY106 | *MATa ade2-1 LYS2 HMRaΔp- (TY1∆-tRNA∆-TY1∆)::pUC - a1* |
| GRY110 | *MATa ade2-1 LYS2 HMRa∆p- (TY1∆-tRNA 300 bp-TY1∆)::pUC - a1* |
| GRY211 | *MATα ADE2 nup2∆::kanMX HMRa∆p- a1-(TY1-tRNA-TY1)* |
| GRY238 | *MATα ADE2 HMRa∆p- a1-(TY1-tRNA-TY1)* |
| GRY266 | *MATa ade2 ppr1∆::kanMX HMR-URA3-(TY1-tRNA-TY1)* |
| GRY323 | *MATa nup60∆::kanMX ade2-1 LYS2 HMRa∆p- (TY1-tRNA-TY1) - a1* |
| GRY325 | *MATa nup60∆::kanMX ade2-1 LYS2 HMRa∆p- (TY1∆-tRNA∆-TY1∆)::pUC - a1* |
| GRY704 | *MATa nup60∆::kanMX ade2-1 LYS2 HMRa∆p- (TY1∆-tRNA 300 bp-TY1∆)::pUC - a1* |
| PSY2946 | *MATa NUP2-9xMyc(C-term)::TRP1 ade2* |
| GRY356 | *MATa NUP2-9xMyc(C-term)::TRP1 ade2 HMR-tRNA∆* |
| GRY400 | *MATa nup2∆::kanMX ade2-1 LYS2 HMRa∆p- (TY1-tRNA-TY1) - a1* |
| GRY402 | *MATa nup2∆::kanMX ade2-1 LYS2 HMRa∆p- (TY1∆-tRNA∆-TY1∆)::pUC - a1* |
| GRY414 | *MATa nup2∆::kanMX ade2-1 LYS2 HMRa∆p- (TY1∆-tRNA 300 bp-TY1∆)::pUC - a1* |
| PSY2825 | *MATa NUP60-9xMyc(C-term)::TRP1 ade2* |
| GRY512 | *MATa NUP60*-9xMyc(C-term)::*TRP1 ade2* *HMR-tRNA∆* |
| GRY291 | *MATα ade2 ppr1Δ::kanMX HMRE-URA3-HMRI* |
| GRY575 | *MATα ade2 nup2∆::kanMX ppr1Δ::kanMX HMRE-URA3-HMRI* |
| GRY684 | *MATα ade2 sir3∆::HIS3 ppr1Δ::kanMX HMRE-URA3-HMRI* |
| GRY686 | *MATα ade2 nup60∆::HIS3 ppr1Δ::kanMX HMRE-URA3-HMRI* |
| GRY687 | *MATα ade2 esc1∆::kanMX ku70∆::Hph ppr1Δ::kanMX HMRE-URA3-HMRI* |
| GRY264 | *MATα ade2 ppr1∆::kanMX HMRI-URA3-tRNA^Thr^* |
| GRY541 | *MATa ade2 ppr1∆::kanMX nup2∆::kanMX HMRI-URA3-tRNA^Thr^* |
| GRY685 | *MATα ade2 ppr1∆::kanMX nup60∆::HIS3 HMRI-URA3-tRNA^Thr^* |
| GRY683 | *MATα ade2 ppr1∆::kanMX sir3∆::HIS3 HMRI-URA3-tRNA^Thr^* |
| GRY691 | *MATα ade2 ppr1∆::kanMX esc1∆::kanMX ku70∆::Hph HMRI-URA3-tRNA^Thr^* |
| GRY630 | *MATα LacI-GFP::ADE2 RECR::LEU2 HMR-GIT1-256xLacO::TRP1 esc1∆::kanMX ku70∆::Hph YIPLac-HDEL-dsRED::natMX ura3 LYS2* |
| GRY632 | *MATα LacI-GFP::ADE2 RECR::LEU2 HMR-GIT1-256xLacO::TRP1 nup60∆::HIS3 esc1∆::kanMX ku70∆::Hph YIPLac-HDEL-dsRED::natMX ura3 LYS2* |
| GRY636 | *MATa LacI-GFP::ADE2 RECR::LEU2 HMR-GIT1-256xLacO::TRP1 YIPLac-HDEL-dsRED::natMX ura3 his3* |
| GRY701 | *MATa LacI-GFP::ADE2 RECR::LEU2 HMR-GIT1-256xLacO::TRP1 nup60∆::HIS3 YIPLac-HDEL-dsRED::natMX ura3* |
| GRY716 | *MATa NUP60*-9xMyc(C-term)::*TRP1* *ade2* *sir4Δ::HIS3* |
| GRY567 | *MATα NUP60*-9xMyc(C-term)::*TRP1* *esc1Δ::kanMX ku70Δ::Hph ura3 ade2* *LYS2* |
| JRY4806 | *MATα ade2 HMR-E::ss(5G-E-B)-HMR-∆I* |
| TM47 | *MATα ADE2 lys2 HMR-E::ss(5G-E-B)-HMR-I::ss(B-5G) trp1* |
| TM58 | *MATα HMR-E::ss(5G-E-B)-HMR-I trp1* |
| TM69 | *MATα HMR-E::ss(5G-E-B)-HMR-I::ss(B-5G) ADE2 LYS2 trp1 sir3∆::LEU2* |
| TM82 | *MATα HMR-E::ss(5G-E-B)-HMR-I::ss(B-5G) ade2 LYS2 trp1 esc1∆::kanMX ku70∆::Hph* |
| SLJ2594 | *MATa* *NUP49*-GFP *his3*::GFP-LacI::*HIS3* *ARS607*::256xLacop-4xlexAop::*TRP1* |
